# Why do lipid nanoparticles target the liver? Understanding of biodistribution and liver-specific tropism

**DOI:** 10.1016/j.omtm.2025.101436

**Published:** 2025-02-15

**Authors:** Mahboubeh Hosseini-Kharat, Kristen E. Bremmell, Clive A. Prestidge

**Affiliations:** 1 Clinical and Health Sciences, Centre for Pharmaceutical Innovation, University of South Australia, Adelaide, SA 5000, Australia

**Keywords:** lipid nanoparticles, LNPs, liver targeting, apolipoprotein E, ApoE, biodistribution, therapeutic delivery

## Abstract

Lipid nanoparticles (LNPs) are now highly effective transporters of nucleic acids to the liver. This liver-specificity is largely due to their association with certain serum proteins, most notably apolipoprotein E (ApoE), which directs them to liver cells by binding to the low-density lipoprotein (LDL) receptors on hepatocytes. The liver’s distinct anatomy, with its various specialized cell types, also influences how LNPs are taken up from the circulation, cleared, and how effective they are in delivering treatments. In this review, we consider factors that facilitate LNP’s effective liver targeting and explore the latest advances in liver-targeted LNP technologies. Understanding how LNPs are targeted to the liver can help for effective design and optimization of nanoparticle-based therapies. Comprehension of the cellular interaction and biodistribution of LNPs not only leads to better treatments for liver diseases but also delivers insight for directing nanoparticles to other tissues, potentially broadening their range of therapeutic applications.

## Graphical abstract

**Figure:**
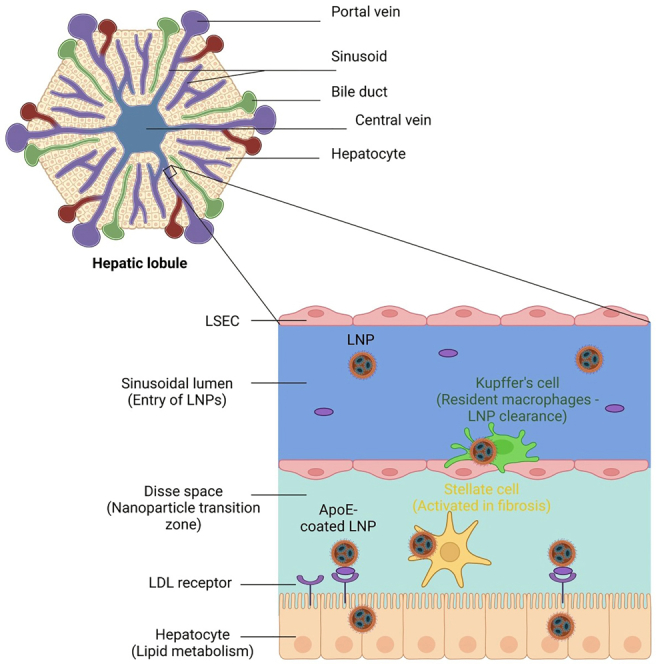

## Teaser

Lipid nanoparticles (LNPs) preferentially accumulate in the liver through ApoE-mediated LDL receptor binding. This review examines this specificity and explores strategies to enhance targeting, expanding LNP applications beyond hepatic diseases.

## Introduction

Lipid nanoparticles (LNPs) are an important delivery vehicle for therapeutic molecules—most notably nucleic acids—as they can encapsulate and shield them from degradation.^1^^,^^2^^,^^3^^,^^4^ LNP technology has come a long way, starting with early liposomal drug systems and advancing to sophisticated formulations designed for precise nucleic acid delivery. Today, LNPs stand as a promising clinically advanced drug delivery system, illustrated by Food and Drug Administration-approved therapies such as Onpattro for hereditary transthyretin-mediated amyloidosis and mRNA vaccines for COVID-19, underscoring their transformative impact on modern medicine.^5^^,^^6^^,^^7^^,^^8^^,^^9^

One key feature of LNPs is their preferential accumulation in the liver, a phenomenon that has been well exploited for treating liver disease. This liver-specific tropism depends on interactions with serum proteins (e.g., apolipoprotein E [ApoE]) as well as the liver’s anatomy and physiology.^10^^,^^11^ Upon systemic administration, LNPs rapidly bind to ApoE, which facilitates their targeting to hepatocytes via low-density lipoprotein (LDL) receptor.^12^ As an additional driver of LNP accumulation, the liver’s participation in the reticuloendothelial system (RES) is fundamental to ApoE-mediated targeting.^10^ Kupffer cells, the liver’s resident macrophages that filter nanoparticles from the bloodstream, contribute to liver tropism but can also reduce therapeutic efficacy by trapping LNPs before they reach hepatocytes.^13^ To address this limitation, lipid compositions are optimized to evade macrophage uptake or to maximize delivery to hepatocytes.^14^

The role of the RES in LNP biodistribution is exemplified by Wang et al.,^15^ who demonstrated how hard nanomaterials are sequestered in the liver through interactions with its microarchitecture and specific cellular components. They have shown that liver-resident Kupffer cells, B cells, and endothelial cells are responsible for nanomaterial accumulation, with Kupffer cells having the highest uptake. At the same time, nanomaterials enter the liver sinusoids at a slower flow rate than in systemic circulation, which increases their probability of uptake by these phagocytic cells, such as Kupffer cells.

**Figure 1:**
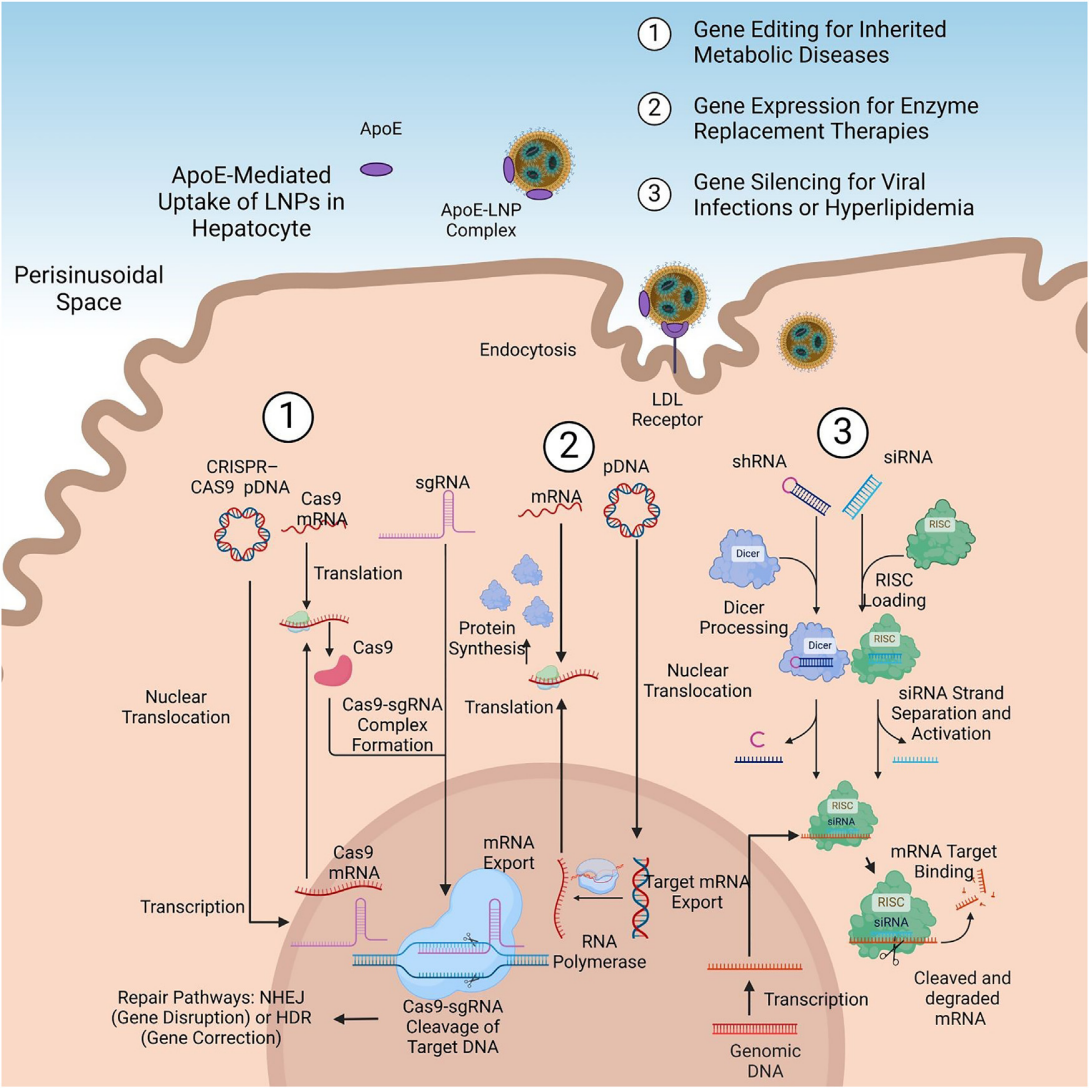
Schematic representation of intracellular pathways of LNPs in liver-targeted therapies LNPs, guided to hepatocytes via ApoE-LDL receptor interactions, follow three main therapeutic applications (1) Gene Editing: CRISPR-Cas9 systems delivered as plasmid DNA (pDNA) or mRNA enable DNA repair through non-homologous end-joining (NHEJ) or homology-directed repair (HDR), addressing genetic disorders; (2) Gene Expression: mRNA or pDNA drives therapeutic protein production for enzyme replacement therapies; and (3) Gene Silencing: siRNA or short hairpin RNA (shRNA) mediates mRNA degradation, silencing genes involved in metabolic or infectious diseases.^17^ Created with BioRender.com. RISC, RNA-induced silencing complex.

Once inside hepatocytes, LNPs enable applications such as gene editing, gene expression, and gene silencing^16^ (Figure 1), as previously reported.^17^ Their physicochemical attributes—such as size, surface charge, and lipid composition—are key in shaping their intracellular pathways and optimizing biodistribution efficiency.^18^ For example, ionizable lipids with specific acid dissociation constants (pKa) improve endosomal escape by optimizing the charge interactions within the endosome.^19^ The pKa values of ionizable lipids cause them to remain neutral at physiological pH and reduce off-target effects. These lipids become positively charged in the acidic endosomal environment.^20^ This protonation disrupts the endosomal membrane by inducing a phase transition from the lamellar phase to a non-lamellar inverted hexagonal phase, which releases the payload into the cytosol.^21^ The proton sponge effect also further helps this process by increasing osmotic pressure and rupturing the endosome.^20^ By binding to ApoE, ionizable lipids help guide LNPs to hepatocytes via LDL receptors.^22^ The lipid composition can also be tailored to improve ApoE interaction and maximize uptake efficiency.^23^

Recent studies (e.g., those using high-throughput screening) have identified formulations optimized for liver delivery, and techniques such as quartz crystal microbalance with dissipation monitoring (QCM-D) have delivered essential data on lipid-protein interactions for ApoE-mediated targeting.^24^ These developments have improved our understanding of how LNP compositions influence ApoE interactions and biodistribution pathways. Developments in LNP design, e.g., using selective organ targeting (SORT) molecules, have improved tissue specificity. By modifying lipid composition, SORT molecules help target organs beyond the liver, expanding LNP-based therapies. SORT molecules are specific lipids added as a fifth component to conventional four-component LNPs. The chemical structure of these molecules determines organ specificity: ionizable lipids enhance liver targeting, anionic lipids direct LNPs to the spleen, and permanent cationic lipids with quaternary ammonium groups increase lung targeting. SORT molecules function through interaction with specific plasma proteins after PEG-lipid shedding. The adsorbed plasma proteins enable receptor-mediated uptake in target organs.^25^^,^^26^

In this review, we examine how LNPs interact specifically with the liver, focusing on liver-specific pathways (e.g., ApoE-LDL receptor interactions, endosomal escape).^12^ Distinct from other reviews, we explore how understanding the liver-specific pathways and mechanisms of LNPs can inform the development of LNP-based therapies for other organs and diseases by identifying common physiological processes or targeting strategies.^25^^,^^27^

## Interactions between liver cell types and LNPs

**Figure 2:**
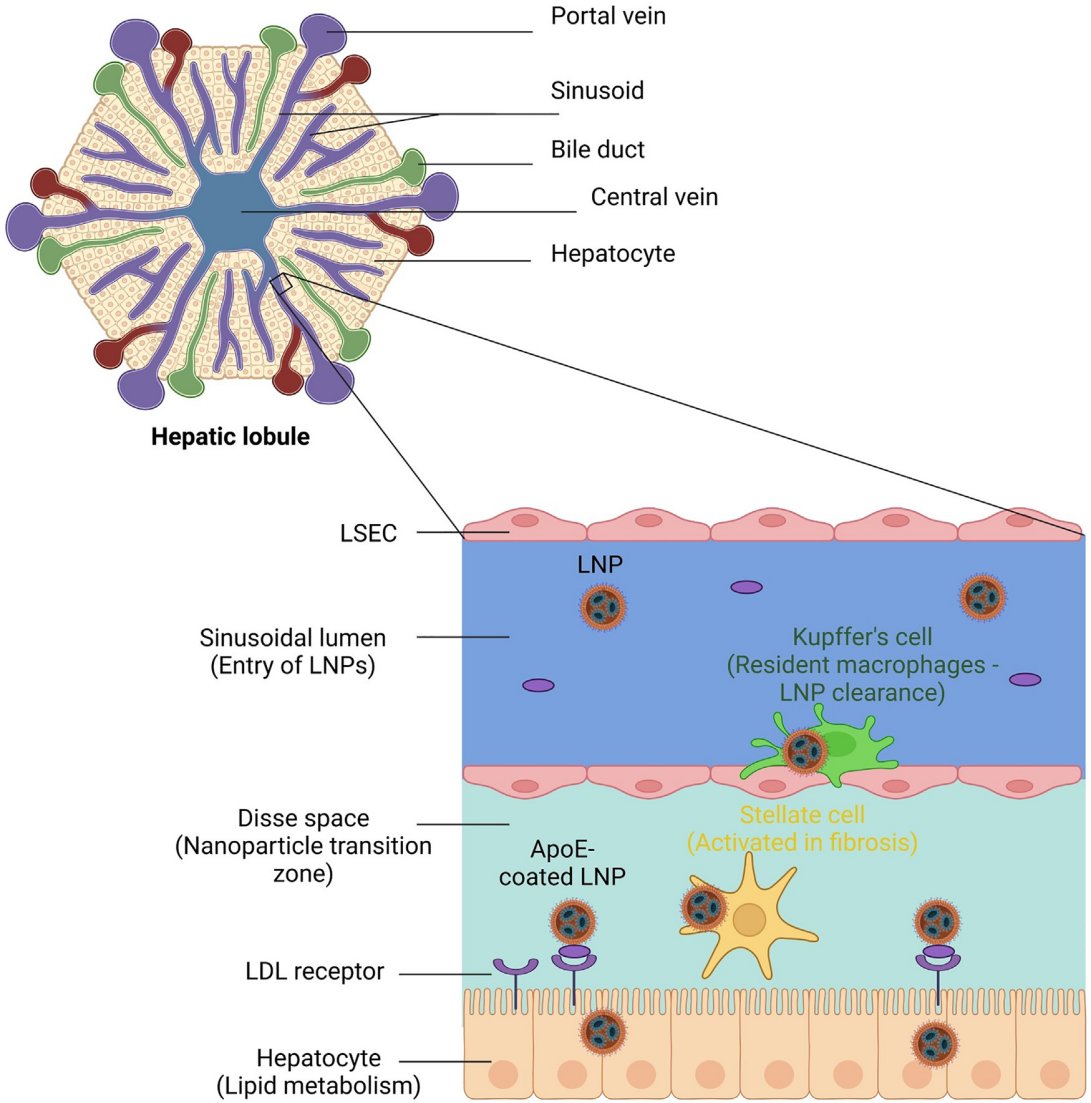
Schematic representation of LNP uptake in the liver (Top) The hepatic lobule structure with a zoomed-in region (sinusoidal organization). (Bottom) Magnified view of LNP trafficking and cellular interactions in the liver microenvironment. Kupffer cells and stellate cells clear some LNPs, while successful LNPs pass through the sinusoidal lumen, traverse fenestrated LSECs, and become ApoE-coated in the space of Disse before reaching hepatocytes via receptor-mediated endocytosis. Created with BioRender.com.

Liver cells help metabolism, immune surveillance, and nanoparticle processing. Hepatocytes, Kupffer cells, liver sinusoidal endothelial cells (LSECs), and hepatic stellate cells (HSCs) are the main cell types that compose the liver.^28^^,^^29^^,^^30^^,^^31^ Of particular importance, these cells interact with LNPs and influence their biodistribution and functional delivery. Figure 2 shows a schematic overview of LNP uptake in the liver, demonstrating the journey of LNPs through the sinusoidal lumen, across LSECs and the Disse space, to hepatocytes, where receptor-mediated endocytosis occurs. The interactions of LNPs with other liver cell subtypes, such as CD74-expressing macrophages and CD32-expressing endothelial cells, affect how efficiently nucleic acids are delivered.^32^ Table 1 summarizes these cell types and their interactions with LNPs.

**Table 1:** Key liver cell types and their interactions with LNPs

Liver cell type	Description	Key characteristics	Ref(s)
Kupffer cells	Specialized macrophages in liver sinusoids; clear nanoparticles and foreign particles via phagocytosis.	Rapid LNP uptake; includes inflammatory (CD74High) and tolerogenic (CD74Low) subtypes.	Kim et al.; Sago et al.; Chen et al.^23^^,^^32^^,^^33^
Hepatocytes	Main liver cells for protein synthesis, detoxification, and lipid metabolism; crucial in LNP uptake.	Efficient LNP uptake via LDL receptor; smaller LNPs (<100 nm) permeate easily.	Kim et al. and MacParland et al.^23^^,^^34^
LSECs	Fenestrated endothelial cells that facilitate nanoparticle transfer between blood and hepatocytes.	Pores (100–140 nm) make LSECs permeable to small nanoparticles; fenestrated structure.	MacParland et al.^34^
Stellate cells and other non-parenchymal cells	Store vitamin A, regulate fibrosis, and interact minimally with LNPs; role in liver therapy.	Modulate LNP responses in fibrosis or cirrhosis; potential therapeutic targets in liver disease.	MacParland et al.^34^

Kupffer cells are distinct macrophages that reside in the liver sinusoids, are responsible for a large fraction of the body’s total macrophages, and are involved in clearing circulating nanoparticles and other foreign materials from the blood. Kupffer cells rapidly take up LNPs, resulting in their predominant distribution in the liver after intravenous administration.^23^^,^^33^ Alongside their phagocytic function, Kupffer cells have defined subpopulations with inflammatory (CD74High) and tolerogenic (CD74Low) phenotypes,^32^ which can influence the immune response and nanoparticle clearance. Their high uptake often limits the amount of LNPs reaching hepatocytes, as Kupffer cells sequester the nanoparticles before they can reach their target cells, reducing functional delivery.^35^

He et al.^11^ demonstrated that modulating Kupffer cell activity can enhance nanoparticle delivery to target tissues. Depletion of Kupffer cells has been shown to improve the functional delivery of nanoparticles to hepatocytes and improve therapeutic outcomes, especially in cancer models.^36^

Key strategies include:

(1) Saturation of Kupffer cells using non-toxic materials to temporarily reduce their phagocytic capacity. Liposomes, colloidal carbon, and fat emulsions are examples of materials used to saturate Kupffer cells, allowing therapeutic nanoparticles to circulate longer before being sequestered.^11^
(2) Inhibition of endocytic pathways through pharmacological agents that block nanoparticle uptake. Examples of such agents are chloroquine, gadolinium chloride, and cytochalasin B, which inhibit pathways such as clathrin-mediated or scavenger receptor-mediated endocytosis, reducing nanoparticle clearance.^11^^,^^37^
(3) Depletion of Kupffer cells to minimize liver sequestration and enhance nanoparticle circulation to target tissues. For example, clodronate liposomes are used to selectively target macrophages, resulting in their depletion. While effective, this approach needs to be carefully examined because it can affect the immune system and may lead to risks such as splenomegaly.^38^^,^^39^^,^^40^

Hepatocytes are the main parenchymal cells of the liver, responsible for liver-specific metabolic functions, including the synthesis of proteins (e.g., albumin), detoxification, and lipid metabolism. Because of interactions with LDL receptors, these cells are highly permeable to LNPs, especially those smaller than 100 nm in diameter. ApoE-coated LNPs bind to LDL receptors and allow LNP uptake into hepatocytes.^23^ The heterogeneity of hepatocytes is also remarkable; they exhibit zonation across the liver acinus, with different metabolic roles depending on their location.^34^ Hepatocyte zonation influences LNP behavior. Zone 1 (periportal) hepatocytes perform oxidative metabolism, while Zone 3 (pericentral) hepatocytes specialize in detoxification and lipid metabolism. Zone 3 hepatocytes enhance ApoE-mediated LNP uptake through higher LDL receptor expression.^23^ Smaller LNPs (<100 nm) use the fenestrations in LSECs to target Zone 3, while the proximity of Zone 1 to blood flow facilitates rapid uptake.^30^^,^^31^ Therapeutic designs should consider the specific functions of hepatocyte zonation: for example, targeting Zone 3 for detoxification therapies due to its role in lipid and xenobiotic detoxification, and Zone 1 for metabolic disorders because of its focus on oxidative metabolism.^41^ Moreover, variations in ploidy among hepatocytes are vital in their spatial and functional specialization. This adds another dimension to the complexity of liver zonation, shaping critical metabolic processes, for example, lipid metabolism and the clearance of nanoparticles.^34^^,^^42^

LSECs are highly specified endothelial cells that line the liver sinusoids. Unlike other endothelial cells, LSECs are fenestrated, which allows them to facilitate the transfer of molecules, including LNPs, into the space of Disse, where hepatocytes reside. These fenestrae, small pores around 100–140 nm in diameter, make LSECs highly permeable to small nanoparticles.^34^ Once in proximity to hepatocytes, LNPs that successfully bind to ApoE can be taken up via LDL receptors, promoting functional delivery. ApoE is essential for LNPs to target the liver. In the bloodstream, ApoE binds to LNPs, forming a protein corona that allows them to be recognized by LDL receptors on hepatocytes.^22^^,^^43^ The lipid composition of LNPs (e.g., ionizable and helper lipids) also influences ApoE binding efficiency.^22^^,^^44^ In addition, the efficiency of ApoE-coated LNP uptake is influenced by hepatocyte zonation, as metabolic and cellular differences across liver zones determine the fate and distribution of LNPs.^16^

However, LNPs that fail to bind ApoE or that become coated with other serum proteins may be redirected to non-target cells or cleared by the spleen.^24^^,^^36^ LSECs also express a variety of receptors, including scavenger receptors (e.g., stabilin-1 and stabilin-2)^45^ and mannose receptors,^23^ that can mediate the active uptake of functionalized LNPs. For example, mannose-modified LNPs can selectively bind LSECs, improving RNA therapeutic delivery.^23^ In addition, the slow blood flow in liver sinusoids allows more time for LNPs to interact with LSECs.^15^ Finally, altering the size, charge, and surface chemistry of LNPs can optimize their interaction with LSECs, with smaller particles (<100 nm) more readily translocated through the fenestrae and internalized.^23^ Given their high endocytic capacity and strategic location in liver sinusoids, LSECs are essential to the liver’s clearance of circulating LNPs.

**Figure 3:**
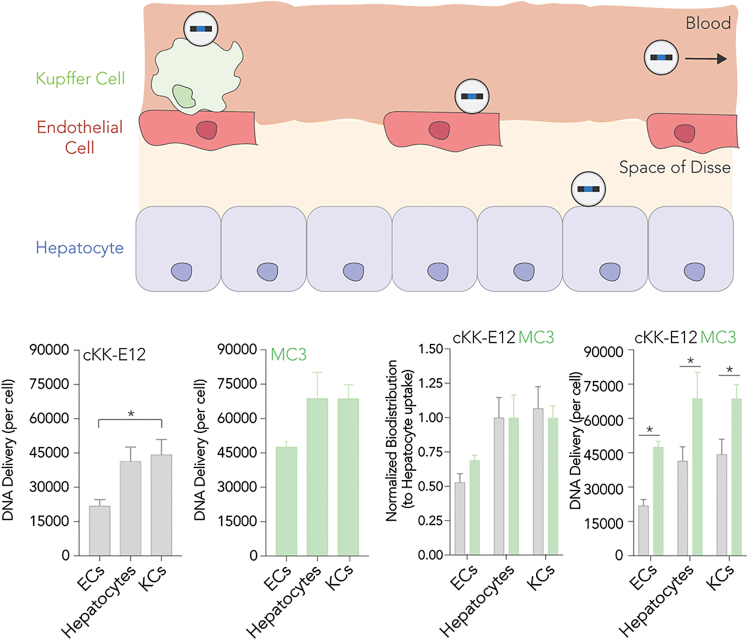
Biodistribution of LNPs formulated with either of the lipids MC3 (a clinically approved ionizable lipid) and cKK-E12 (an ionizable lipid with a multi-tail structure) in the liver microenvironment (Top) As LNPs travel through the liver, they interact with Kupffer cells (KCs), endothelial cells (ECs), and hepatocytes. (Bottom) From left to right: cKK-E12-mediated DNA delivery to ECs, hepatocytes, and KCs; MC3-mediated DNA delivery to ECs, hepatocytes, and KCs; normalized DNA delivery (relative to hepatocytes) by cKK-E12 and MC3, showing similar intrahepatic biodistribution patterns; and a comparison of DNA delivery by cKK-E12 and MC3 across major liver cell types. MC3^46^ and cKK-E12^47^^,^^48^, adapted from^32^. *p* < 0.05, One-way ANOVA and Two-way ANOVA with Tukey’s Multiple Comparison. Error bars represent standard error. Copyright © 2019, Biomedical Engineering Society.

Sago et al.^32^ investigated how specific liver cell subtypes—Kupffer cells, liver endothelial cells, and hepatocytes—interacted with LNPs. LNPs accumulated broadly across these cell types, though functional mRNA delivery was notably higher in liver endothelial cells than in Kupffer cells or hepatocytes. Liver endothelial cells, with their distinctive discontinuous vasculature, act as a gateway, allowing LNPs to extravasate into the space of Disse, where they can interact with hepatocytes and other liver cells. Notably, endothelial cell subtypes, for example, CD32High (central venous zone) and CD32Low (periportal zone), showed different interactions with LNPs, with CD32Low cells exhibiting higher accumulation. This spatial distribution and receptor expression in liver endothelial cells influenced LNP uptake and processing, which in turn impacted biodistribution and functional delivery (Figure 3).

Liver stellate cells (which store vitamin A and contribute to fibrosis) and cholangiocytes (which help produce bile) have important roles but interact with LNPs less than hepatocytes and Kupffer cells. Still, stellate cells contribute to liver function and the response to nanoparticles.^49^^,^^50^^,^^51^ As part of the fibrotic matrix in liver disease, they may also influence how LNPs interact with the immune system.^34^

## LNP composition and liver targeting

LNPs are effective in targeting the liver; however, to understand their localization, it is important to distinguish biodistribution from functional delivery. This distinction helps clarify how LNPs can target specific organs such as the liver while guaranteeing the therapeutic cargo is effectively delivered in the target cells.^32^^,^^52^ Biodistribution is the process of LNPs accumulating in specific tissues after entering circulation, while functional delivery depends on the successful release and action of the therapeutic cargo (e.g., mRNA or small interfering RNA [siRNA]) in target cells.^53^ This distinction is important because LNPs can accumulate in tissues such as the liver without producing intended therapeutic effects.

Functional delivery may be impaired by factors such as inadequate endosomal escape or protein corona,^54^ which can drive LNP accumulation in non-target cells while hindering cellular uptake and effective intracellular delivery in target cells. Proteomic analyses have shown that the composition of the protein corona can affect nanoparticle-cell interactions, altering both the distribution and efficacy of the delivery system by influencing receptor recognition and endocytosis mechanisms.^55^ Functional delivery is also impacted by challenges in intracellular trafficking and cargo release once inside cells. Even after cellular uptake, therapeutic molecules must navigate the complex intracellular environment to reach their site of action, whether that be the cytoplasm for mRNA translation or specific organelles for other therapeutics.^56^^,^^57^^,^^58^

Several studies have demonstrated rapid liver accumulation of LNPs within minutes of administration, largely due to the liver’s extensive blood flow and porous endothelium.^24^^,^^35^^,^^59^ However, this high level of accumulation does not directly translate to efficient functional delivery. Recent work by Lam et al.^60^ showed that while LNPs of different sizes can accumulate similarly in the liver, their functional delivery to hepatocytes varies between species. In non-human primates, smaller LNPs (50–60 nm) showed approximately 5-fold higher protein expression in liver compared with conventional larger particles (70–80 nm). More importantly, both formulations exhibited similar pharmacokinetic profiles and liver accumulation. This finding demonstrates that the liver microarchitecture, for example, the size of liver fenestrae, which are smaller in primates than rodents, is important for efficient delivery. This result is further supported by Sato et al.,^61^ who showed that uptake mechanisms in hepatocytes vary based on ionizable lipid structure. They further showed that the apparent pKa of the ionizable lipid influences intrahepatic LNP distribution, which can be tuned by modifying the chemical structure around a tertiary amine. LNP-based siRNA therapies also show that while LNPs accumulate in the liver, gene silencing depends on factors such as dosing, lipid composition, and cell interactions.^62^ For example, inefficient endosomal escape can limit delivery despite favorable biodistribution.

Different LNP formulations show variability in functional delivery despite similar levels of biodistribution. For example, LNPs formulated with sphingomyelin or DSPC exhibited different transfection efficiencies in hepatocytes compared with other liver cells.^59^ The structure of ionizable lipids, in particular the head group and tail (carbon chain) length, is an important factor driving both liver uptake and functional delivery. For example, lipids such as DLin-MC3-DMA with a pKa in the range of 6.2–6.5 have been shown to be optimized for liver-specific gene silencing in hepatocytes.^12^ Recent research found that biodegradable ketal-ester lipids, used as ionizable lipids, made delivery more efficient while reducing liver toxicity. Their rapid breakdown in the liver also improved safety.^63^ Couture-Senécal et al.^64^ reported how the chemical structure of ionizable lipids influences their hydrolysis rates, modifying biodegradability and liver retention. With cell-free hydrolysis assays involving lipases and base reactions, they found that lipids with sterically accessible ester bonds, e.g., LP-01 and SM-102, were rapidly hydrolyzed and cleared from the liver. In contrast, more sterically hindered lipids, e.g., DLin-MC3-DMA and ALC-0315, exhibited slower degradation, resulting in prolonged liver retention.

Another example is the use of SORT nanoparticles to enhance targeted mRNA delivery to organs, such as the liver, spleen, and lungs.^65^ By adding a fifth “SORT molecule” (e.g., 18PA for spleen targeting and DOTAP for lung targeting) to the conventional four-component LNP, modifications in chemical composition have been shown to affect biodistribution. For liver-specific targeting, the addition of an ionizable cationic lipid, e.g., DODAP, has been shown to enhance liver accumulation. The SORT LNP mechanism uses serum protein adsorption, where proteins (e.g., ApoE) can bind to the nanoparticle surface and mediate uptake by target cells, such as hepatocytes via LDL receptors or through other tissue-specific pathways, depending on the recruited proteins.

**Figure 4:**
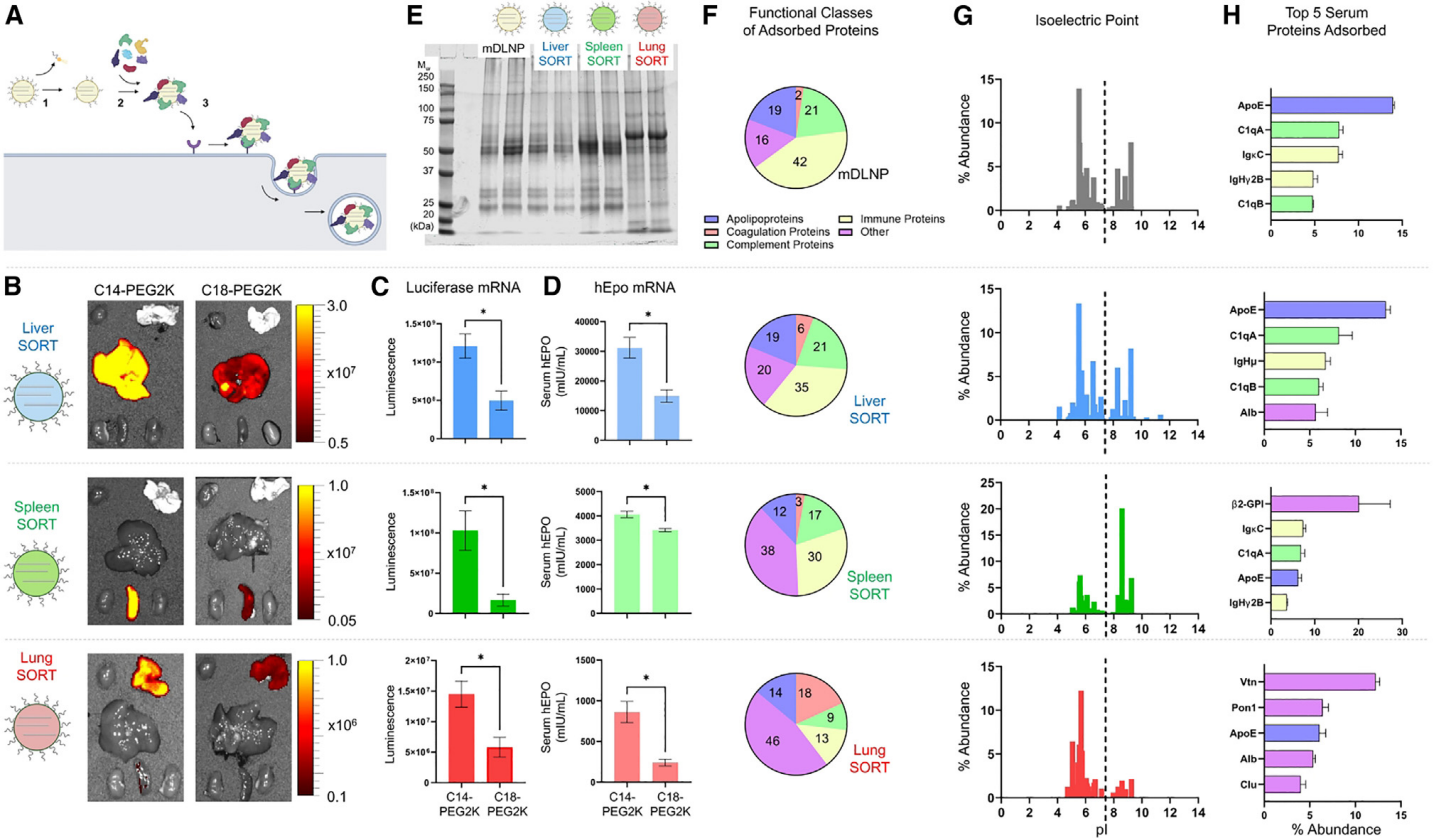
Mechanism of SORT LNP tissue targeting (A) Proposed targeting mechanism. (B) *Ex vivo* bioluminescence in major organs of C57BL/6 mice injected with liver, spleen, or lung SORT LNPs using either sheddable (C14-PEG2K) or less-sheddable PEG lipids (C18-PEG2K), showing reduced luminescence with less-sheddable PEG. (C) Quantified luminescence from FLuc mRNA in target organs. (D) ELISA of serum hEPO post-injection with hEPO mRNA SORT LNPs, showing less potency with less-sheddable PEG. (E–H) Plasma protein binding profiles on SORT LNPs for different organs, illustrating how SORT molecule structure affects protein corona composition and isoelectric point distribution, impacting targeting specificity. Data: mean ± SEM; unpaired two-tailed Student’s t test, *p* < 0.05.^65^ © 2021 National Academy of Sciences.

The mechanism of tissue-specific targeting of SORT LNPs is illustrated in Figure 4. This proposed three-step consists of (1) desorption of PEG lipids from the nanoparticle surface, (2) binding of specific plasma proteins to the exposed SORT molecules, and (3) receptor-mediated uptake in target tissues. The figure shows how modifying the PEG-lipid properties (e.g., using less-sheddable PEG lipids like C18-PEG2K) significantly reduces targeting efficiency, as demonstrated by decreased bioluminescence in target organs. Additionally, the plasma protein binding profiles shown in the figure emphasize how the molecular structure of SORT molecules influences the protein corona composition and isoelectric point distribution, directly affecting organ-specific biodistribution.^65^ SORT nanoparticles also can be designed to selectively edit therapeutically relevant cell types such as epithelial cells, endothelial cells, B cells, T cells, and hepatocytes, using various gene-editing modalities, e.g., mRNA, Cas9 mRNA/single guide RNA, and Cas9 ribonucleoprotein complexes.^66^

These improvements in LNP design and targeting have opened the door to transformative therapeutic applications. A review by D’Alessio et al.^41^ discussed how LNPs can be used for liver-targeted gene therapy in inherited metabolic diseases (IMDs). By naturally accumulating in the liver, LNPs deliver therapeutic mRNA and gene-editing components directly to hepatocytes, helping to detoxify harmful metabolites and address enzyme deficiencies associated with these conditions. This detoxification occurs through the correction of enzyme deficiencies, enabling hepatocytes to process and eliminate toxic substrates that would otherwise accumulate and cause systemic damage.

## Analysis of liver-targeted LNP studies

Key examples from the literature on liver-targeting LNPs are summarized in Table 2. The results showed that intravenous delivery is the most commonly used route, demonstrating its effectiveness in targeting liver cells. Key observations, listed below, show the influence of lipid components, surface chemistry, and target cell interactions.

(1) Ionizable lipids contributed to increased endosomal escape and liver-specific delivery. Lipids, such as MC3 and cKK-E12, had specific pKa values optimized for hepatocyte uptake.^32^^,^^67^ Importantly, tail length affected organ distribution. For example, shorter tails targeted the spleen, and longer tails favored liver uptake.^67^ Formulations such as 5A2-SC8 and 3A5-SC14 showed different targeting behavior; e.g., 5A2-SC8 enhanced ApoE-mediated hepatocyte delivery, while 3A5-SC14 favored Kupffer cell uptake.^36^
(2) Helper lipids, such as DOPE and DSPC, impacted biodistribution. For example, DOPE improved ApoE-mediated liver uptake, while DSPC directed transport to the spleen, showing how fine-tuning LNPs allowed for specific targeting.^23^^,^^24^
(3) Cholesterol stabilizes LNPs and also influences targeting. Esterified cholesterol improved endothelial delivery, while oxidized cholesterol favored Kupffer cells and immune cells, reducing hepatocyte interaction.^68^^,^^69^ Cationic cholesterol further facilitated delivery to both the liver and extrahepatic tissues, e.g., the lungs and heart.^70^
(4) Surface chemistry was important for targeting efficiency. PEG lipids improved circulation, while sheddable variants (e.g., pH-sensitive or enzymatically cleavable PEG) helped target the liver. Mannose-PEG, on the other hand, enhanced receptor-specific delivery to LSECs.^23^ pH-sensitive PEGylated lipids increased the stability of the formulation and enabled efficient liver genome editing.^71^ Siloxane-incorporated lipidoids also improved endosomal escape and liver-specific mRNA delivery.^72^
(5) LNPs proved promising in liver-targeted therapies, enabling applications, e.g., cancer treatment, gene editing, metabolic restoration, and gene silencing. For example, circUGP2 LNPs inhibited tumor progression via p53 activation while partially targeting Kupffer cells.^33^ Cre mRNA-loaded COATSOMESS-OP also achieved precise gene editing in hepatocytes (Cre mRNA encodes the Cre recombinase enzyme).^73^ Biodegradable LNPs delivered ARG1 mRNA for metabolic restoration in arginase deficiency models,^74^ and CL4H6 siRNA-loaded LNPs efficiently silenced hepatocyte genes with high specificity.^75^
(6) Target cell specificity is a requirement for successful delivery. Kupffer cells often sequester LNPs, reducing delivery to hepatocytes. However, depletion strategies have been shown to improve hepatocyte targeting by minimizing LNP clearance by Kupffer cells.^35^ For example, depletion of Kupffer cells was shown to improve nanoparticle delivery to tumors by up to 150-fold, though the maximum tumor accumulation remains only 2% of the injected dose, demonstrating systemic challenges in nanoparticle targeting. LSECs, because of their fenestrations, facilitated selective delivery, such as for receptor-specific LNPs.^23^ Targeted delivery to stellate cells and macrophages reduced fibrosis markers, restored liver function and promoted repair (e.g., tissue regeneration and fibrosis reversal).^76^^,^^77^^,^^78^ Similarly, targeted therapies using hepatocyte growth factor (HGF)/epidermal growth factor (EGF) mRNA and NM-FGF19 mRNA-loaded LNPs successfully treated chronic liver injuries and metabolic dysfunction by delivering therapeutic payloads to specific hepatic cells.^79^^,^^80^ Recent studies demonstrated new mechanisms for liver-targeted delivery. For example, anionic LNPs targeted Kupffer cells and LSECs through stabilin receptors,^45^ while GalNAc-LNPs entered hepatocytes through asialoglycoprotein (ASGP) receptor-mediated uptake, independent of LDL receptor.^81^
(7) Some LNP formulations demonstrated extrahepatic targeting beyond the liver. For example, the presence of egg sphingomyelin (ESM) in LNP formulations improved protein expression in the spleen and bone marrow, in addition to the liver.^59^ (4S)-KEL12 directed LNPs to the spleen over the liver,^63^ while cationic cholesterol enabled delivery to the lungs and heart.^70^ Differences in lipid composition and administration route also affected transfection efficiency, such as higher targeting of the spleen and muscle compared with the liver.^82^

**Table 2:** LNP compositions, therapeutic payloads, administration routes, targeted liver cell types, and key findings

LNP composition	Therapeutic gene/encapsulated element	Route of administration	Targeted liver cells	Key findings	Ref.
MC3 or cKK-E12, cholesterol, PEG-lipid, DSPC	QUANT DNA sequences	Intravenous	Kupffer cells, liver endothelial cells, hepatocytes	LNPs accumulate in Kupffer and endothelial cells; CD74/CD32 markers enhance mRNA delivery in endothelial cells despite lower biodistribution.	Sago et al.^32^
DOTAP, DPPC, folate-conjugated DSPE-PEG2000, cholesterol	circUGP2 plasmid	Tail vein	Tumor cells, Kupffer cells	circUGP2 LNP activates p53, inhibiting ICC progression and targeting tumor cells; partial Kupffer cell uptake.	Chen et al.^33^
DOPE, cholesterol, PEG-lipid, ionizable lipids (241C10 to 246C10)	Firefly luciferase mRNA (mFLuc), Cre mRNA	Intravenous	Hepatocytes, LSECs	ApoE aids hepatocyte uptake; mannose-PEG targets LSECs; particle size and PEG affect targeting.	Kim et al.^23^
MC3, cholesterol, PEG-lipid, DSPC or ESM (40 mol%)	GFP mRNA, luciferase mRNA	Intravenous	Hepatocytes, extrahepatic cells	High ESM in LNPs enhances liver/extrahepatic targeting, prolongs circulation, and boosts protein expression in spleen and bone marrow.	Chander et al.^59^
Gold, silver, silica, liposomes with PEG-coating	Various (chemotherapeutics, fluorescent tags)	Intravenous	Kupffer cells, tumor cells	Kupffer cell depletion enhances tumor nanoparticle delivery by up to 150-fold, with a maximum of 2% accumulation in liver tumors.	Tavares et al.^35^
DOPE or DSPC, cholesterol, PEG-lipid, C12-200	Cy3-siRNA, luciferase mRNA	Intravenous	Liver (hepatocytes), spleen (macrophages)	DOPE-LNPs enhance liver uptake via ApoE binding, while DSPC-LNPs favor spleen targeting, improving siRNA/mRNA delivery.	Zhang et al.^24^
5A2-SC8 or 3A5-SC14 (ionizable lipids differing in alkyl chain length and saturation), DSPC, cholesterol, PEG-DMG	siRNA (siFVII), let-7g miRNA	Intravenous	Hepatocytes, Kupffer cells	5A2-SC8 binds ApoE for targeted hepatocyte delivery, while 3A5-SC14 favors Kupffer cell uptake. Only 5A2-SC8 shows strong therapeutic effects in liver cancer.	Johnson et al.^36^
COATSOME®SS-OP, DOPC, cholesterol, DMG-PEG2000	Cre mRNA	Intravenous (Jugular vein)	Hepatocytes	Efficiently induces liver-specific knockout in floxed mice, with minimal effects on non-target organs.	Morita et al.^73^
Esterified, oxidized, and unmodified cholesterol variants with PEG lipids and 7C1	DNA barcodes, siRNA, sgRNA	Intravenous	Hepatic endothelial cells, hepatocytes	Esterified cholesterol LNPs enhanced liver endothelial cell delivery, while oxidized cholesterol reduced efficiency.	Paunovska et al.^68^
DLin-KC2-DMA, DLin-MC3-DMA, DODAP, cholesterol, DSPC, PEG-DSPE	Nanoluciferase (NLuc) pDNA	Intravenous, Intramuscular	Spleen, muscle, liver	Highest transfection in spleen (IV) and muscle (IM); liver showed moderate transfection based on lipid and route.	Algarni et al.^82^
cKK-E12, cationic cholesterol, PEG-lipid	Cre mRNA	Intravenous	Hepatic cells	Cationic cholesterol in LNPs enhances mRNA delivery to the liver, though lung and heart delivery are predominant.	Radmand et al.^70^
Siloxane-incorporated lipidoids (Si6-C14b)	(FLuc) mRNA	Intravenous	Hepatocytes, Kupffer cells	Si6-C14b enables strong liver-specific mRNA delivery with high expression in liver cells.	Xue et al.^72^
(4S)-KEL12, DLin-MC3-DMA, SM-102, cholesterol, DSPC, DMG-PEG2000	(FLuc) mRNA	Intravenous, Intramuscular	Hepatocytes, Kupffer cells	(4S)-KEL12 favors spleen over liver with lower hepatotoxicity, unlike liver-targeting DLin-MC3-DMA and SM-102.	Lv et al.^63^
Ionizable lipid (various tail lengths), cholesterol, DSPC, DMG-PEG2000	(FLuc) mRNA	Intravenous	Hepatocytes	Short-tail LNPs shifted mRNA expression from liver to spleen, while long-tail LNPs maintained liver targeting.	Hashiba et al.^67^
cKK-E12, DOPE, oxidized cholesterol (20α-OH)	Cre mRNA, DNA barcode	Intravenous	Hepatic endothelial cells, Kupffer cells, immune cells	Oxidized cholesterol enhances delivery of mRNA and DNA to liver immune and endothelial cells, with reduced targeting of hepatocytes.	Paunovska et al.^69^
P13–8Y ionizable lipid, DOPE, cholesterol, PEG-lipid	siGTSE1	Intravenous	Hepatocytes, Kupffer cells	P13–8Y LNPs effectively deliver siGTSE1 to hepatocytes, reducing GTSE1, collagen deposition, and fibrosis markers. This leads to restoration of liver function.	Jeong et al.^76^
Lipid-Protamine-DNA (LPD) nanoparticles, conjugated with aminoethyl anisamide (AEAA)	pDNA encoding Relaxin (RLN), miR-30a-5p	Intravenous	HSCs, macrophages	RLN and miR-30a-5p-loaded LPD nanoparticles promote macrophage phenotype switching to aid in fibrosis resolution, targeting HSCs and reducing fibrosis markers.	Hu et al.^77^
Anisamide-tethered lipidoids (AA-T3A-C12), cholesterol, DSPC, PEG-lipid	siHSP47 RNA	Intravenous	Activated HSCs	AA-T3A-C12 LNP achieved targeted delivery to activated HSCs, achieving 65% HSP47 knockdown and significantly reducing collagen deposition in a liver fibrosis model.	Han et al.^78^
Biodegradable liver-targeted LNPs	Human codon-optimized ARG1 mRNA	Intravenous	Hepatocytes	LNPs delivered ARG1 mRNA to liver, restoring urea cycle and normalizing ammonia/arginine in arginase deficiency model.	Truong et al.^74^
pH-sensitive PEGylated and cationic lipids (DOTAP, DLin, DOPE, cholesterol)	iGeoCas9 RNP	Intravenous	Hepatocytes, macrophages, endothelial cells	Achieved liver genome editing with 37% efficiency, showing high stability and targeting potential for both liver and lung.	Chen et al.^71^
Proprietary ionizable lipid, phosphatidylcholine, cholesterol, PEG-lipid	HGF and EGF mRNA	Intravenous	Hepatocytes, Kupffer cells, and endothelial cells	HGF/EGF mRNA-LNP promotes liver repair, reducing steatosis and restoring alanine aminotransferase in chronic injury models.	Rizvi et al.^79^
Ionizable lipid, fusogenic lipid, structural lipid, PEG lipid	NM-FGF19 mRNA	Intravenous	Hepatocytes	NM-FGF19 mRNA-LNP reduced liver steatosis, improved cholesterol levels, and modulated bile acids in an MASH (Metabolic Associated Steatohepatitis) model.	Lopez-Pascual et al.^80^
CL4H6, cholesterol, PEG-lipid	siRNA	Intravenous	Hepatocytes	CL4H6-LNPs enabled effective siRNA delivery to hepatocytes with high gene silencing and low toxicity.	Sato et al.^75^
Anionic LNPs (DSPG, cholesterol, PEG-lipid, DSPC)	mRNA	Intravenous	LSECs, Kupffer cells	Preferentially target hepatic RES; efficient mRNA delivery via stabilin pathways.	Pattipeiluhu et al.^45^
GalNAc-lipid LNPs	CRISPR base editing therapy (ANGPTL3)	Intravenous	Hepatocytes	Achieve LDL receptor-independent liver delivery with high editing efficiency and durability.	Kasiewicz et al.^81^

## Liver and extrahepatic LNP barriers

### Non-specific uptake

Sequestration of LNPs by Kupffer cells and LSECs often limits the fraction of nanoparticles reaching hepatocytes, the primary therapeutic target for many hepatic diseases. Strategies to prevent non-specific uptake, such as transient depletion of Kupffer cells (typically achieved using clodronate liposomes), remain experimentally successful but lack clinical feasibility due to potential immune suppression risks.^32^^,^^40^^,^^83^

### Challenges in active targeting

Active targeting of LNPs encounters several limitations rooted in the complex nature of biological systems.^25^ For example, receptors, such as the ASGP receptor, commonly used for hepatocyte delivery, naturally bind glycoproteins. This binding creates competition that decreases nanoparticle binding efficiency. Further to this, receptor expression varies between individuals and is often altered in diseased states, such as liver fibrosis or hepatocellular carcinoma, making precise targeting even more difficult.^84^ When nanoparticles enter the bloodstream, they quickly acquire a protein corona—a layer of adsorbed proteins—that can obscure targeting ligands, reducing specificity and increasing off-target effects.^85^^,^^86^ High doses of nanoparticles may also saturate receptors, further limiting their ability to appropriately reach target cells.

### Impact of liver zonation

Variability in metabolic activity and receptor distribution (e.g., differences in enzyme expression and transporter density) across heterogeneous liver zones complicates uniform nanoparticle delivery. Current designs often fail to address liver lobule variability due to variations, e.g., blood flow, metabolism, and receptor distribution, causing uneven drug distribution.^11^^,^^26^^,^^87^

### Barriers in diseased liver states

Pathophysiological remodeling in diseased livers, such as the loss of fenestrations in sinusoidal endothelial cells and the deposition of extracellular matrix in liver fibrosis, hinders nanoparticle extravasation and cellular uptake. These changes require new designs that can either penetrate fibrotic tissue or use alternative delivery routes.^88^^,^^89^^,^^90^

### Safety and ethical challenges

As LNP-based therapies advance, it is vital to address ethical and long-term concerns. Main concerns include informed consent, fair access, and the ethical limitations of gene editing (e.g., CRISPR-Cas9).^91^^,^^92^ Researchers continue to study immune responses, toxicity, and off-target effects (e.g., repeated dosing).^93^^,^^94^ PEGylated LNPs may cause hypersensitivity reactions or immune responses with repeated use, demonstrating the importance of long-term safety studies.^95^^,^^96^ Additionally, the environmental impact of large-scale LNP production and disposal should be evaluated.^97^

### Challenges in achieving extrahepatic delivery

LNP formulations’ strong preference for liver uptake poses a significant barrier to treating diseases requiring delivery to other tissues or systemic distribution. Adding to this challenge is the formation of a protein corona during circulation, which can obscure targeting ligands and further limit the capacity of LNPs to bypass the liver and reach non-hepatic targets. Resolving this imbalance is critical to unlocking the broader therapeutic applications of LNP technologies.^98^^,^^99^

## Future perspectives

The future of LNP technology for liver targeting lies in overcoming existing limitations through new design and a greater insight of liver biology. A potential direction is the development of materials that respond dynamically to the liver microenvironment. By engineering nanoparticles to release their therapeutic cargo in response to specific triggers—such as pH changes or enzymatic activity—developments aim to enhance delivery precision and reduce off-target effects. Such smart nanocarrier systems would allow for more controlled therapeutic release, e.g., in heterogeneous diseased tissues like fibrotic or cancerous livers.^27^^,^^100^ Developments in LNP design, such as bioinspired and biomimetic nanoparticles, are improving functional delivery. For example, cell membrane-coated nanoparticles mimic red or immune cells, enhancing circulation, immune evasion, and targeting.^101^

Another important focus is the personalization of nanoparticle formulations. Leveraging advances in genomics and proteomics, researchers can design LNPs personalized for an individual’s liver physiology or disease state. This strategy could overcome the variability in receptor expression and liver pathology among patients, e.g., differences in ASGP receptor levels, improving the specificity and efficacy of targeted therapies. Personalized LNP systems could also reduce the likelihood of adverse effects, as they would be optimized for each patient’s molecular profile.^102^^,^^103^

Adding multifunctionality to LNPs creates new opportunities for both treatment and diagnosis.^104^ For example, researchers could design nanoparticles with imaging agents and therapeutic payloads, allowing real-time tracking of their distribution and effectiveness. This theragnostic approach, e.g., in personalized medicine or oncology treatments, would improve treatment monitoring, reduce trial-and-error dosing, and help clinicians adjust therapies based on patient response.^103^

## Acknowledgments

M. Hosseini-Kharat acknowledges the support of the 10.13039/501100001787University of South Australia (UniSA) through the Lipid Nanoparticle Research Scholarship and the Research Training Program (RTP) Domestic Fee Offset.

## Author contributions

M.H.-K.: Writing – review & editing, Writing – original draft, Conceptualization. K.E.B.: Writing – review & editing. C.A.P.: Writing – review & editing, Supervision.

## Declaration of interests

The authors declare no competing interests.
